# Great Gifts during Life and after Death

**Published:** 1911-12-30

**Authors:** 


### The Hospital

The Workers' Newspaper of Administrative Medicine and Institutional Life, Administration, National Insurance and Health.

No. 1329, Vol. LI. SATURDAY, DECEMBER 30TH, 1911. PRICE ONE PENNY.

### GREAT GIFTS DURING LIFE AND AFTER DEATH.

One of the most generous givers now living told us that he once spent three months of hard work in trying to persuade several of his wealthy friends to contribute largely in their lifetime to a benevolent object of which they all heartily approved. Sometimes in the evening a friend consented to hand him a cheque in the morning, but when the morning came one after the other confessed they could not bring themselves to part with so large a sum during their lifetime. They all promised him, however, to leave the money as he wished in their wills. Of course, one reason is that many people cannot persuade themselves to give away large sums of money in their lifetime. Exactly why is a problem which is intensified by the fact that in every case we have in mind a gift of this kind would have meant in practice very little more than a book entry. In the case of wealthy business men experience shows that those of them who have the courage to give large sums to charity in their own names have often to face the ungenerous and wanton criticism, that theirs is not noble generosity but a matter of advertisement. There are some natures which are so constituted that they always see a nigger in the fence, whatever may be done by another, or whatever blessings may be poured upon themselves. We have met people who have suddenly and unexpectedly come into a fortune, when their first thought was, what a burden this money will be. Such a habit of mind represents the lowest depth of degrading pessimism.

So those of us who regard it as a high privilege to work continuously for the upkeep of the voluntary hospitals must recognise how great has been the service rendered to our cause and to the hospitals by the founding of King Edward's Hospital Fund. It has won its way to the confidence of the wealthy, and of the managers of all classes of hospitals throughout the United Kingdom. A decision of the King's Fund upon any controversial point is accepted as judicial and authoritative. Such a fact demonstrates convincingly the wisdom which King Edward VII. brought to bear upon every public affair to which he set his mind. King Edward never took anyone into counsel or friendship until he had thoroughly satisfied himself that his personal standard of conduct was of the highest, and that he possessed undoubted pre-eminence by his special ability and knowledge.

King Edward's influence over his friends was equally remarkable, and Sir Ernest Cassel, whom we can personally testify to be one of the most scrupulously upright and generous of men, was no doubt cheered and encouraged by his association with his Majesty. Sir Ernest Cassel has shown the courage which few possess, to give largely to the least objectionable of all charities—institutions for the relief and cure of the sick. Quite apart from his Christmas gift of £50,000 in memory of his daughter, who was one of the most attractive and beautiful of women, he has, by not giving his money anonymously, and by his wisdom in strengthening the King's Fund by entrusting it with the distribution of his gifts to the hospitals, made this £50,000 of the widest possible value. He has shown wealthy men how they can give their money and secure its wise distribution free from the grave dangers which formerly beset the gift of large sums in charity. He has made it plain that he joins hands with the poorest, to whom voluntary hospitals are a safeguard, by upholding the voluntary system of hospitals as the best in the world. So the voluntary hospitals throughout the country, whether they have participated in the present gift or not, must feel grateful to Sir Ernest Cassel, for his example must have the widest possible influence for good upon all classes of generous people throughout the British Empire. He has shown that to give largely and wisely during one's lifetime can be made the most fruitful means of ensuring that such money will do the maximum of good.

